# Targeting High-Risk Neuroblastoma Patient-Derived Xenografts with Oncolytic Virotherapy

**DOI:** 10.3390/cancers14030762

**Published:** 2022-02-01

**Authors:** Colin H. Quinn, Andee M. Beierle, Sara Claire Hutchins, Raoud Marayati, Laura V. Bownes, Jerry E. Stewart, Hooper R. Markert, Michael H. Erwin, Jamie M. Aye, Karina J. Yoon, Gregory K. Friedman, Christopher D. Willey, James M. Markert, Elizabeth A. Beierle

**Affiliations:** 1 Division of Pediatric Surgery, Department of Surgery, University of Alabama at Birmingham, Birmingham, AL 35205, USA; chquinn@uab.edu (C.H.Q.); rmarayati@uabmc.edu (R.M.); lbownes@uabmc.edu (L.V.B.); jessy@uab.edu (J.E.S.); hmarkert95@gmail.com (H.R.M.); mhe0004@uab.edu (M.H.E.); 2 Department of Radiation Oncology, University of Alabama at Birmingham, Birmingham, AL 35233, USA; abeierle@uab.edu (A.M.B.); cwilley@uabmc.edu (C.D.W.); 3 Division of Pediatric Hematology Oncology, Department of Pediatrics, University of Alabama at Birmingham, Birmingham, AL 35233, USA; schutchins@uabmc.edu (S.C.H.); jaye@uabmc.edu (J.M.A.); gfriedman@uabmc.edu (G.K.F.); 4 Department of Pharmacology and Toxicology, University of Alabama at Birmingham, Birmingham, AL 35233, USA; kjyoon@uab.edu; 5 Department of Neurosurgery, University of Alabama at Birmingham, Birmingham, AL 35233, USA; jmarkert@uabmc.edu

**Keywords:** neuroblastoma, oncolytic viruses, three-dimensional printing, immunotherapy, patient-derived xenografts

## Abstract

**Simple Summary:**

More than half of children with high-risk neuroblastoma will not survive despite aggressive treatment regimens. The discovery of suitable novel therapies that target high-risk disease remain limited. Oncolytic virotherapy employs common viruses, such as herpes simplex virus. These viruses are modified to solely target cancer cells and avoid damage to healthy cells. The previous work of others and ourselves has shown oncolytic herpes simplex viruses (oHSVs) are effective in targeting cultured neuroblastoma cancer cell lines, but to our knowledge, no one has demonstrated the effects of oHSV in neuroblastoma patient-derived xenograft cells. Herein, we show that oHSV works well in high-risk neuroblastoma patient-derived xenografts and provide critical preclinical evidence to translate this therapy to a clinical setting.

**Abstract:**

Cancer is the leading cause of death by disease in children, and over 15% of pediatric cancer-related mortalities are due to neuroblastoma. Current treatment options for neuroblastoma remain suboptimal as they often have significant toxicities, are associated with long-term side effects, and result in disease relapse in over half of children with high-risk disease. There is a dire need for new therapies, and oncolytic viruses may represent an effective solution. Oncolytic viruses attack tumor cells in two ways: direct infection of tumor cells leading to cytolysis, and production of a debris field that stimulates an anti-tumor immune response. Our group has previously shown that M002, an oncolytic herpes simplex virus (oHSV), genetically engineered to express murine interleukin-12 (mIL-12), was effective at targeting and killing long term passage tumor cell lines. In the current study, we investigated M002 in three neuroblastoma patient-derived xenografts (PDXs). PDXs better recapitulate the human condition, and these studies were designed to gather robust data for translation to a clinical trial. We found that all three PDXs expressed viral entry receptors, and that the virus actively replicated in the cells. M002 caused significant tumor cell death in 2D culture and 3D bioprinted tumor models. Finally, the PDXs displayed variable susceptibility to M002, with a more profound effect on high-risk neuroblastoma PDXs compared to low-risk PDX. These findings validate the importance of incorporating PDXs for preclinical testing of oncolytic viral therapeutics and showcase a novel technique, 3D bioprinting, to test therapies in PDXs. Collectively, our data indicate that oHSVs effectively target high-risk neuroblastoma, and support the advancement of this therapy to the clinical setting.

## 1. Introduction

Pediatric cancer is the second leading cause of death in children [1]. Neuroblastoma, a tumor of neural crest origin, is the most common extracranial solid tumor of childhood and is responsible for more than one in eight pediatric cancer deaths. Advances in neuroblastoma treatment have resulted in improved outcomes for children with low-risk disease [2]. However, for patients with high-risk disease, long-term survival remains less than 50% [1,2]. Additionally, current therapies often result in detrimental long-term side effects in those who are cured. Clearly, further research is needed to identify novel and innovative therapies to attack this tumor.

The use of immunotherapies to treat pediatric hematologic malignancies has been successful [3,4], but employing immunotherapy for the treatment of solid tumors remains challenging [5]. For neuroblastoma in particular, the immunosuppressive tumor microenvironment limits the efficacy of these treatments [6]. Oncolytic viruses offer an innovative approach to employ immunotherapy. Oncolytic viruses attack tumors by (i) direct cytolysis as a result of viral replication and (ii) creation of a debris field that activates cytotoxic immune cells. Oncolytic viruses have achieved early clinical success in other pediatric solid tumors with immunosuppressive microenvironments such as glioblastoma [7] and are FDA-approved for use in melanoma [8], but the use of oncolytic herpes simplex viruses (oHSVs) for neuroblastoma remains limited to a single phase I clinical trial (NCT00931931) and failed to progress to phase II [9,10]. The absence of widespread use in pediatric solid tumors is partly due to the lack of a thorough understanding of the therapeutic response to oncolytic viruses and to the heterogeneity of neuroblastoma tumors.

Researchers have been grappling to find cancer models that more closely recapitulate the heterogeneity of cancers seen in the human condition while continuing to provide a rapid and efficient evaluation of potential therapeutics. Patient-derived xenografts (PDXs) are one such model. PDXs are felt to be superior to continuously cultured cells as these cell lines alter their original properties, including phenotypes and genotypes, after being cultured in contrived conditions for long periods of time [11]. Further, cells in culture lack components of the tumor microenvironment that, in the case of immunotherapies, can significantly affect their efficacy [12]. PDXs for pediatric solid tumors do have some limitations, such as poor engraftment rates and slow, inconsistent growth [13]. Thus, we employed 3D bioprinting technology to complement our in vitro studies of oHSV and serve as a surrogate to in vivo studies. Lastly, we and others have previously demonstrated that findings from in vitro studies with PDX cells were not always replicated in the in vivo PDX model [14]. Thus, it is critical to continue to explore and develop models that will help examine therapeutic effectiveness in a more reproducible environment.

M002 is an oHSV that has been genetically engineered with a deletion in the γ_1_34.5 gene. This gene deletion renders the virus aneurovirulent, allowing it to replicate only in cancer cells. M002 is uniquely armed with murine interleukin (mIL)-12, designed to stimulate a host immune response [15]. Our laboratory, in conjunction with our collaborators, has generated robust pre-clinical evidence that this unique oHSV functions to kill tumor cells in several pediatric solid tumors [16,17,18,19,20,21], including neuroblastoma [22]. The previous studies in neuroblastoma were limited to long-term passage cell lines, and there has yet to be an investigation of M002 in neuroblastoma PDX. Thus, in the current studies, we aimed to investigate the effects of M002 on human neuroblastoma PDXs.

## 2. Materials and Methods

### 2.1. Cell Lines and Culture

Vero cells and human neuroblastoma PDX cell lines were cultured in sterile incubators at 37 °C with 5% carbon dioxide (CO_2_). Vero cells were obtained from the American Type Culture Collection (ATCC; CCL-81, Manassas, VA, USA) and cultured in modified Eagle’s medium (MEM, Gibco, Thermo Fisher Scientific Inc., Waltham, MA, USA) with 7% fetal bovine serum (FBS, Hyclone, Suwanee, GA, USA), 2 µM l-glutamine (Thermo Fisher Scientific Inc.), and 1 µg/mL penicillin/streptomycin (Gibco, Thermo Fisher Scientific Inc.). For all PDXs used in this study (COA3, COA6, and COA129), dissociated tumor cells were cultured in neurobasal (NB) medium (Life Technologies, Carlsbad, CA, USA) supplemented with B-27 without Vitamin A (Life Technologies), N2 (Life Technologies), amphotericin B (250 μg/mL, Thermo Fisher Scientific Inc.), gentamicin (50 μg/mL, Thermo Fisher Scientific Inc.), l-glutamine (2 mM, Thermo Fisher Scientific Inc.), epidermal growth factor (10 ng/mL, Miltenyi Biotec, San Diego, CA, USA), and fibroblast growth factor (10 ng/mL, Miltenyi Biotec). For cell quantification, single cell suspensions were created using trypsin with 1% EDTA (Gibco, Thermo Fisher Scientific Inc.) for adherent cells and accutase (Gibco, Thermo Fisher Scientific Inc.) for cells in suspension. Cells were then allowed to incubate in either accutase or trypsin for 10 min prior to centrifugation and resuspension in their respective medias. A 20 μL sample of the cells was mixed at a 1:1 ratio with trypan blue (Gibco, Thermo Fisher Scientific Inc.) for dead cell detection, and subsequent cell counts were performed using a hemocytometer.

### 2.2. Establishing Patient-Derived Xenografts

All PDXs were established as previously described [23] using patient samples from Children’s Hospital of Alabama under University of Alabama at Birmingham (UAB) Institutional Review Board-approved protocol (IRB 130627006). Tumors identified in patients and used in this study for experimentation include two high-risk neuroblastomas (COA3 and COA6) and a low-risk metastatic special (MS) neuroblastoma (COA129) that originated from a diverse group of patients. Briefly, following written informed consent of each patient’s guardian and assent from each patient as appropriate, a fresh tumor specimen was obtained from surgical excision and temporarily placed in serum-free Roswell Park Memorial Institute 1640 (RPMI, 30–2001, ATCC) medium. The tumor specimen was separated and allocated as follows: (i) formalin fixed for paraffin embedding; (ii) placed in liquid nitrogen for storage at −80 °C; and (iii) placed in sterile media for implantation. Female NOD SCID mice (Envigo, Prattville, AL) were used for implantation following a UAB Institution Animal Care and Use Committee (IACUC) protocol, (IACUC-009186). Briefly, mice were anesthetized with 3% inhalational isoflurane, and 16 mm^3^ tumor pieces were placed into the subcutaneous space of the animals’ flanks. Animals were housed in a pathogen-free environment while being monitored routinely for overall health and tumor growth through weekly tumor volume measurements. Tumor volumes were calculated using the formula (width^2^ × length)/2, and when volumes met IACUC parameters, tumors were harvested. Tumors were allocated as described above with an additional piece reserved for tumor dissociation and in vitro experimentation. Tumors were dissociated using a Tumor Dissociation Kit (Miltenyi Biotec) per manufacturer’s protocol and cultured as described above in NB media.

### 2.3. Virus

These studies used a genetically modified oHSV, M002, which has been extensively described [15]. This oncolytic virus is a herpes simplex virus (HSV)-1 F strain with a deletion in the native thymidine kinase locus as well as a thymidine kinase gene inserted into deleted regions of both γ_1_34.5 loci. There was an additional insertion of the murine IL-12 (mIL-12) subunits that lies under the transcriptional control of the murine Early-growth response-1 promoter (Egr-1). There are two copies of the entire construct present, with a single copy inserted into each of the γ_1_34.5 loci. The virus will replicate and produce mIL-12 when the native thymidine kinase gene is restored [15]. Titer of M002 was completed as previously described [16]. Briefly, Vero cells (1.5 × 10^5^ cells per well) were plated in 24-well plates and allowed 24 h to attach and form a confluent monolayer. Ten-fold dilutions of stock virus in infection medium (% FBS and 1% pen/strep in EMEM (30-2006, ATCC)) were applied to the Vero cells for 2 h, then the inoculum was removed and the plates were washed with media. After an additional 48 h of incubation, May–Grunwald stain (Sigma Aldrich, Millipore Sigma, St. Louis, MO, USA) in methanol was applied for 20 min and plates were washed and allowed to dry overnight. Plaques were counted and the titer was calculated and reported as plaque-forming units per milliliter (PFU/mL).

### 2.4. Flow Cytometry

Flow cytometry assessed the cell surface expression of the receptors responsible for M002 viral entry including: CD111 (poliovirus receptor-related 1, PVRL1, nectin-1), CD112 (nectin-2), syndecan, and Herpes Virus Entry Mediator (HVEM). PDX cells (10^6^) were cultured overnight in 6-well non-adherent plates. Cells were harvested and centrifuged at 1200 rpm for 4 min. Cell pellets were washed and resuspended in autoMACS^®^ running buffer (130-091-221, Miltenyi Biotec). Suspensions were blocked with 20 µL FcR Blocker (120-000-442, Miltenyi Biotec) for 15 min on ice. Cells only given blocker served as negative controls. After 15 min, 10 μL of phycoerythrin (PE) conjugated anti-human CD111 antibody (Miltenyi Biotec), PE anti-human CD112 antibody (Miltenyi Biotec), allophycocyanin (APC) conjugated anti-human syndecan (Miltenyi Biotec), or APC anti-human HVEM (Miltenyi Biotec), were added for 20 min on ice in the dark. The stained cells were centrifuged, washed twice with autoMACS^®^ buffer (Miltenyi Biotec), and reconstituted in 200 μL of sterile phosphate buffered saline (PBS) for analysis via Attune Next Su™ Flow Cytometer (Thermo Fisher Scientific). Data were analyzed with FlowJo v10.0.6 (Tree Star Inc., Ashland, OR, USA) and represented at least three biologic replicates.

### 2.5. Viral Replication

Viral recovery experiments were performed as described [18]. Briefly, for single step studies, that assess a single growth cycle of the virus, neuroblastoma PDX cells (3 × 10^5^) were infected at a multiplicity of infection (MOI) of 10 PFU/cell for 2 h in 200 μL of infectivity media (EMEM (ATCC) with 1% FBS (Hyclone) and 1% pen/strep (Gibco)) and shaken on a rocker in an incubator at 37 °C with 5% CO_2_. Following infection, cells were spun down and resuspended in 1 mL of NB media (Life Technologies). At 12- and 24-h post-infection, the cells were harvested by adding equal volume of sterile milk and freezing at −80 °C for at least 24 h. Multi-step viral recovery experiments were performed to assess viral replication over multiple growth cycles of the virus. For multi-step studies, PDX cells (3 × 10^5^) were infected at a MOI of 0.1 PFU/cell for 2 h in 200 μL of infectivity media (EMEM (ATCC) with 1% FBS (Hyclone) and 1% pen/strep (Gibco)) and shaken on a rocker in an incubator at 37 °C with 5% CO_2_. Following infection, cells were spun down and resuspended in 1 mL of NB media (Life Technologies). At 6-, 12-, 24-, 48-, and 72-h post-infection, the cells and media were harvested by adding equal volume of sterile milk and freezing at −80 °C for at least 24 h.

To determine the titer of progeny virions for both assays, plates were thawed at 37 °C and sonicated for 30 s. Samples were flash frozen, thawed, and sonicated for two more cycles. Serial dilutions of the milk stocks were made (from 10^0^–10^12^) in infectivity media. Vero cells (1.2 × 10^5^) were grown to confluence in 24 well plates. Milk stock dilutions (200 μL) were added to the Vero cell monolayers. Plates were rocked for 2 h in an incubator at 37 °C with 5% CO_2_, and then the inoculum was removed and replaced with Vero cell media. Following 48 h of incubation, May–Grunwald stain in methanol was applied for 30 min and plates were washed with distilled water for 10 min. Plates were allowed to dry overnight, plaques were counted, and the titer was calculated and reported as PFU/cell.

### 2.6. ELISA

PDX cells (1.5 × 10^4^) were plated in 96-well plates in triplicate for 24 h. Cells were treated with M002 at an MOI proven to adequately infect cells (0, 1 PFU/cell) for 24, 48, and 72 h. At each time point, plates were centrifuged, and the media collected and flash frozen. Production of murine IL-12 was quantified from the flash frozen samples using a total murine IL-12 ELISA kit (Thermoscientific, Rockford, IL, USA) according to manufacturer’s protocol.

### 2.7. Cytotoxicity

COA3 and COA6 cells (1.5 × 10^4^) and COA129 cells (3.0 × 10^4^) were plated in 96-well plates. Cells were treated with increasing MOI of M002 (0.0, 0.01, 0.05, 0.1, 0.5, 1.0 PFU/cell). Following 24, 48, and 72 h of infection, a colorimetric assay was used to determine viability by adding 10 μL of sterile alamarBlue^®^ dye (Invitrogen Life Technologies, Grand Island, NY, USA) to each well and reading absorbance at 542 and 595 nm using a kinetic microplate reader (BioTek Plate Reader, Gen5, BioTek Instruments, Winooski, VT, USA). Measurements were used to estimate the number of PFU to kill 50% of the cells by comparing the reduction in color with the reduction seen in the control group (100% viability). Experiments were repeated with at least three biologic replicates and data reported as fold change ± standard error of the mean (SEM).

### 2.8. Antibodies

The following antibodies were used: rabbit monoclonal anti-Stat1 (Cell Signaling Technologies); mouse monoclonal β-actin (A1978) from Sigma Aldrich; and polyclonal rabbit anti-HSV from Biogenex (PU084-UP, Fremont, CA, USA).

### 2.9. Immunoblotting

PDX cells (2 × 10^6^) were plated in non-adherent conditions in NB media with increasing MOI of M002 (0, 0.1, 1.0 PFU/cell). At 24, 48, and 72 h, cells were washed with PBS, centrifuged, and flash frozen with liquid nitrogen. Whole cell lysates were made using radioimmunoprecipitation (RIPA) buffer (10 mmol/L Tris base pH 7.2, 150 mmol/L NaCl, 1% Na-deoxycholate, 1% Triton X-100, 0.1% sodium dodecyl sulfate (SDS)) supplemented with phenylmethanesulfonylfluoride (PMSF, Sigma), protease inhibitor (Sigma), and phosphatase inhibitor (Sigma). Protein concentrations of the whole cell lysates were determined with a Micro BCA Protein Assay Kit (Thermo Fisher Scientific Inc.), and equal amounts of proteins were loaded for gel electrophoresis. Proteins were mixed with 10 μL of 4× SDS and then separated on SDS polyacrylamide (SDS-PAGE) gels via electrophoresis. A Precision Plus Protein Kaleidoscope molecular weight marker (Bio-Rad, Hercules, CA, USA) was loaded adjacent to samples for protein kDa reference. Gels were transferred to a blotting membrane using Trans-Blot Turbo Transfer System (Bio-Rad) per manufacturer’s protocol. All immunoblots were washed in 1× tris-buffered saline, 0.1% Tween 20 (TBST) buffer. Antibodies were used per manufacturers’ instructions and immunoblots developed with either Immobilon Classico or Crescendo Western HRP Substrate (EMD Millipore). Immunoblots were stripped with stripping buffer (Bio-Rad) at 65 °C for 15 min prior to re-probing. β-actin or GADPH was used to confirm equal protein loading.

### 2.10. RNA Sequencing

COA3, COA6, and COA129, cells (10^7^) were collected and an RNeasy kit (Qiagen, Hilden, Germany) was used to extract RNA per manufacturer’s instructions. Whole cell RNA sequencing was performed by the UAB Center for Genomic Studies. Fragments per kilobase of exon per million mapped fragments (FPKM) values were reported.

### 2.11. Bioprinted Microtumors

After PDX tumor dissociation and suspension in NB media for 24 h, COA6 and COA129 cells (10^8^) were mixed in 1000 μL of bioink composed of 1% sodium alginate and 6% gelatin (Provona^®^ UV-MVG, Dupont Nutrition Norge As, Sandvika, Norway). The homogenous mixture was loaded into a 3 mL Cellink (Cellink, Boston, MA, USA) printing syringe. A Cellink BIO X™ printer (Cellink) was set to print at a pressure of 10 kPa for a 1.2 s extrusion time through a 22-gauge needle. Individual prints were approximately 0.1 cm^3^ and printed into a 5 μM pore Transwell^®^ (Corning Life Sciences, Tewksbury, MA, USA) insert in a 24-well plate. Calcium chloride (100 μL) was added to each print for five minutes to achieve crosslinking. The crosslinking agent was washed from the prints with 500 μL of sterile PBS and prints placed in 2 mL of NB media. Prints were incubated at 37 °C in 5% CO_2_ for five days to form microtumors. Media was replaced daily.

After 5 days, microtumors were directly injected with M002 (10^7^ PFU/10 μL, n = 4) or an equal volume of PBS (n = 4, control) using a 30.5 gauge needle and syringe. Media surrounding the prints was collected, flash frozen, and replaced every day over a course of one week. At the conclusion of the experiment, microtumors were stained with calcein AM (1:1000, Thermo Fisher Scientific) for 30 min and imaged with a Cytation 5 Imaging Reader (BioTek) to assess viability. Images were analyzed using ImageJ software (Ver. 1.49, http://imagej.nih.gov/ij, 15 December 2021) and reported as mean integrated density ± SEM. Tumors were fixed in 80% ethanol for immunohistochemistry and media was utilized in multistep assays to assess titer of progeny virions in the pseudo-tumor microenvironment.

### 2.12. Immunohistochemistry

Bioprinted microtumors were fixed in 80% ethanol and embedded into paraffin for sectioning. Sections of 6 μM were placed onto positive slides and processed as previously described [23]. The primary antibody, anti-HSV1 (1:100, PU084-UP, BioGenex), was added and incubated overnight in a humidity chamber at 4 °C. The slides were washed with PBS, and rabbit secondary antibody (R.T.U. biotinylated universal antibody, Vector Laboratories, Burlingame, CA, USA) was added for 30 min at 22 °C. Staining reaction was applied for 30 min at room temperature with VECTASTAIN Elite ABC reagent (PK-7100, Vector Laboratories) and Metal Enhanced DAB Substrate (Thermo Fisher Scientific) for 2 min. All slides were counterstained with hematoxylin. For each microtumor, there was a negative control (rabbit IgG, 1 µg/mL, EMD Millipore).

### 2.13. Genomic Analyses

The R2: Genomic Analysis and Genomic Database Platform (https://r2.amc.nl, 8 November 2021) was used to compare the expression of STAT1 across different categories of neuroblastoma. The SEQC publicly available database was used as a reference. A one-way ANOVA test was done to determine statistical significance.

### 2.14. Data Analysis

Experiments were repeated with at least three biologic replicates and data reported as mean ± standard error of the mean (SEM). To determine statistical significance, a Student’s *t*-test or ANOVA was used, with *p* ≤ 0.05 considered statistically significant.

## 3. Results

### 3.1. Expression of Viral Entry Mediated Receptors

**Figure 1:**
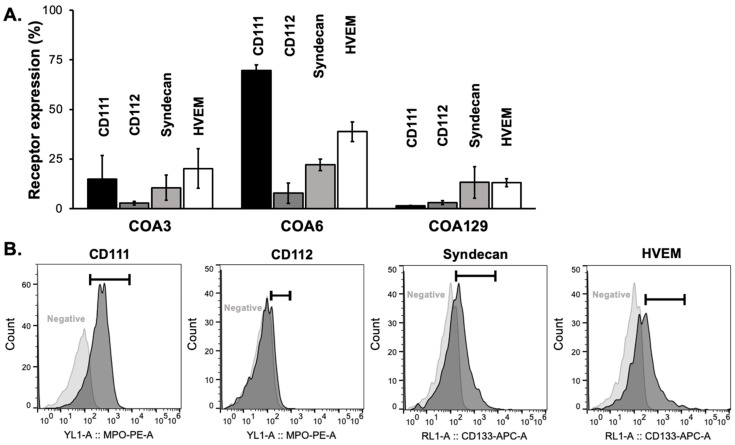
Neuroblastoma PDXs express viral entry receptors. (**A**) Flow cytometry was used to detect the cell surface expression of the four main HSV receptors: CD111, CD112, syndecan, and HVEM in COA3, COA6, and COA129 human neuroblastoma PDX cells. All PDXs expressed all four receptors. (**B**) Representative histograms of CD111, CD112, syndecan, and HVEM cell surface expression and negative controls of COA6 are shown. Data reported as mean ± SEM and represent at least three biologic replicates.

The expressions of viral entry mediated receptors increase cell susceptibility to infection by HSV-1. The four primary HSV-1 receptors are CD111 (anti-PVRL1, nectin-1) [24], CD112 (nectin-2) [25], syndecan (CD138, heparin sulfate proteoglycan) [26], and HVEM [27], with CD111 considered to be the most important [28]. These receptors are functional when present at the cellular membrane and thus are best detected via flow cytometry. We probed COA3, COA6, and COA129 neuroblastoma PDX cells with anti-human CD111, CD112, syndecan, and HVEM and found that each PDX expressed all four viral receptors (Figure 1A,B).

### 3.2. M002 Replication

**Figure 2:**
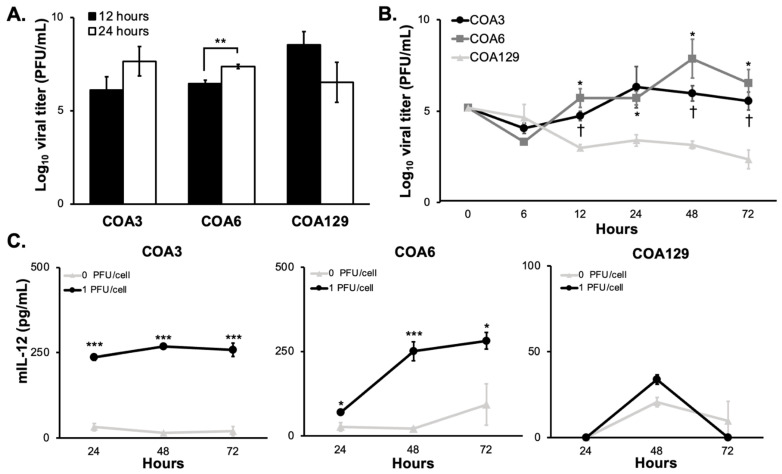
M002 infects and replicates in neuroblastoma PDXs. (**A**) The ability of M002 to replicate in each of the PDX tumors was assessed using a single step assay. COA3, COA6, and COA129 were infected with M002 at an MOI of 10 PFU/cell, and cells and supernates were collected at 12 and 24 h post infection. Viral titers were then determined via Vero monolayers. Viral titer increased over time in the COA3 and COA6 tumor cells, but not in the COA129 (** *p* ≤ 0.01, 12 h vs. 24 h). (**B**) Viral replication rate was then assessed using a multi-step assay. COA3, COA6, and COA129 were infected with M002 at an MOI of 0.1 PFU/cell cells and supernates (due to the non-adherent conditions) were collected at 6, 12, 24, 48, and 72 h. Viral titers were then determined via Vero monolayers. At 12, 48, and 72 h, COA3 and COA6 had significantly higher viral replication than that seen in COA129. Additionally, the rate of M002 replication in the COA129 decreased over time, while it tended to increase in the COA3 and COA6 PDXs. († *p* ≤ 0.05 COA3 vs. COA129, * *p* ≤ 0.05 COA6 vs. COA129). Replication rates between COA3 and COA6 were not significantly different. (**C**) ELISA was used to detect mIL-12. PDXs were treated with M002 (0, 1.0 PFU/cell) and the supernates collected at 24, 48, and 72 h. There was significant production of mIL-12 in both the COA3 (left) and COA6 (middle), but nominal amounts in the COA129 (right) PDX tumor cells (* *p* ≤ 0.05, ** *p* ≤ 0.01, *** *p* ≤ 0.001, compared to 0 PFU/cell). Data are reported as mean ± SEM and represent at least three biologic replicates. A Student’s *t*-test was used for statistical analysis.

In vitro replication of M002 was investigated using single- and multi-step viral recovery experiments. For the single-step studies, neuroblastoma PDX cells were infected with a MOI of 10 PFU/cell. By 12 h post-infection, there were substantial viral titers present in all three PDX cell lines (Figure 2A). Viral titers significantly increased at 24 h in the COA6 while they decreased at 24 h in the COA129. For multi-step experiments, PDX cells were infected with M002 at MOI of 0.1 PFU/cell. At 6, 24, 48, and 72 h post-infection, viral replication was determined using plaque assays as previously described. At 6 h post infection, all three PDXs had a viral titer approximately 10^4^ PFU/mL (Figure 2B). Both COA3 and COA6 maintained viral titers that were significantly higher than that of COA129 at 12, 48, and 72 h.

### 3.3. mIL-12

M002 is genetically engineered to produce mIL-12, a cytokine shown to stimulate an antitumor Th1 immune response, during replication [29]. Therefore, measuring the production of mIL-12 is another method that may be utilized to document viral infection and replication in the PDX cells. PDX cells were infected with M002 (0, 1.0 PFU/cell) and the surrounding media collected at 24, 48, and 72 h post infection. There was a significant increase in the production of mIL-12 over 72 h at 1.0 PFU/cell (259 ± 19 pg/mL, *p* = 5.15 × 10^-4^) in COA3 (Figure 2C, left panel) as well as in COA6 (281 ± 24 pg/mL, *p* = 0.046, Figure 2C, middle panel). In COA129, there was limited production of mIL-12 at all timepoints (Figure 2C, right panel). Such quantities of mIL-12 from the COA3 and COA6 have been shown to generate an immune response in a murine sarcoma model and suggest potential for an immune cell attack on COA3 and COA6 [30]. These data indicate that M002 replicated better in the COA3 and COA6 PDXs than in the COA129 PDX and recapitulated the findings of the multi-step viral recovery experiments (Figure 2B).

### 3.4. Cytotoxicity

**Figure 3:**
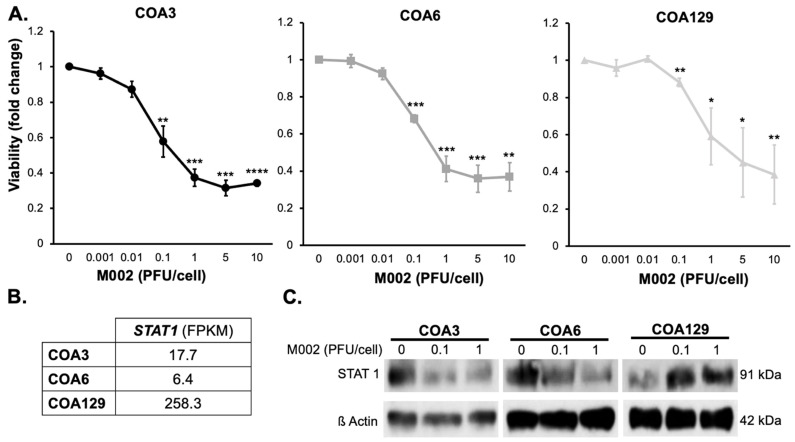
M002 decreased neuroblastoma PDX viability and affected STAT1 expression. (**A**) COA3, COA6, and COA129 were treated with increasing MOIs of M002 (0, 0.001, 0.01, 1, 5, 10) for 72 h and cell viability was measured with alamarBlue^®^ assay. There was a significant decrease in viability in COA3 (left), COA6 (middle), and COA129 (right) with M002 treatment (* *p ≤* 0.05, ** *p ≤* 0.01, *** *p ≤* 0.001, **** *p ≤* 0.0001, compared to 0 PFU/cell). (**B**) RNA abundance of *STAT1* was examined with sequencing in untreated COA3, COA6, and COA129 cells. There was more *STAT1* RNA in COA129 cells. (**C**) STAT1 expression was assessed following M002 infection. COA3, COA6, and COA129 cells (10^6^) were treated with increasing MOI of M002 (0, 0.1, 1) for 72 h. STAT1 expression was analyzed via immunoblotting (separate blots for each PDX). There was a decrease in STAT1 expression in COA3 (left) and COA6 (middle), but an increase in STAT1 in COA129 (right) following infection with M002. Full Western blot scans provided and densitometry values calculated for each blot (Figure S1) Data are reported as mean ± SEM and represent at least three biologic replicates. A Student’s *t*-test was used for statistical analysis (Figure S2).

The objective for oncolytic virotherapy is tumor cell cytotoxicity. The primary mechanism through which M002 kills cells is by cellular burst from the lytic cycle. Since we showed that M002 effectively replicated in the neuroblastoma PDX cells, we measured the effects of M002 infection on viability. COA3, 6, and 129 cells were treated for 24, 48, or 72 h with M002 at increasing MOI (0–10 PFU/cell) and viability measured with alamarBlue^®^ assays. M002 treatment decreased viability of all three PDXs significantly (Figure 3A). The MOI of the virus that resulted in 50% of cell death for the COA3 cells was 0.50 PFU/cell, for the COA6 cells was 0.94 PFU/cell and for the COA129 cells was 3.96 PFU/cell.

### 3.5. M002 Effects STAT1 Expression

Cells have intrinsic anti-viral mechanisms, one of which is the STAT1 signaling pathway. STAT1 responds to the recognition of virus by toll-like receptors and has been shown to halt viral replication [31,32,33], which impedes the efficacy of an oHSV [34]. We investigated baseline levels of *STAT1* RNA in each of the untreated PDXs (Figure 3B). COA129 had more *STAT1* RNA at baseline compared to COA3 or COA6. We then examined STAT1 expression following treatment with increasing MOIs of M002 (0, 0.1, 1 PFU/cell) at 72 h on three separate immunoblots (due to the intensity of STAT1 expression at baseline in the COA129) and found that in the COA3 and COA6 PDX cells, STAT1 decreased with M002 infection (Figure 3C, Figure S1), but in COA129 cells, STAT1 expression increased (Figure 3C, Figure S1).

### 3.6. D Bioprinted PDX Tumors Respond to M002 Infection

**Figure 4:**
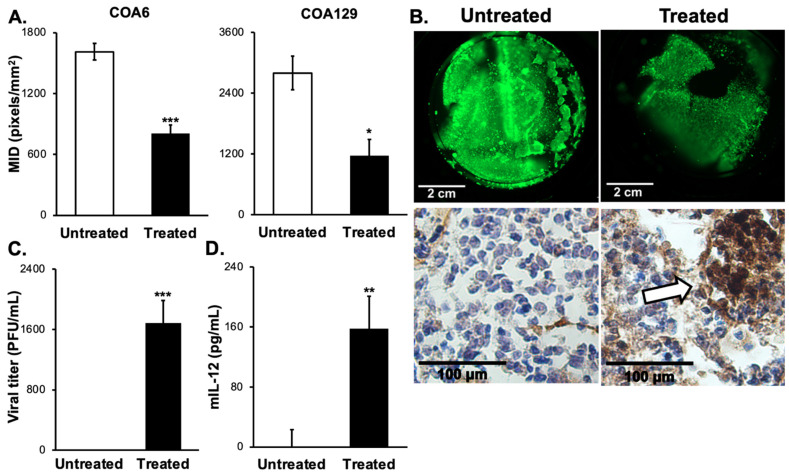
M002 effectively infected, replicated in, and killed neuroblastoma PDX cells ex vivo. (**A**) 3D printed tumors of a high-risk neuroblastoma PDX (COA6) and low-risk MS neuroblastoma (CO129) were used. COA6 and COA129 microtumors were given intratumoral injections of M002 (n = 4, MOI 10^7^/10 μL in sterile PBS) or control (n = 4, 10 μL of sterile PBS). Following one week of infection, tumors were stained with calcein AM to detect viable tumor tissue (viability indicated by green, fluorescent staining) and analyzed with ImageJ to determine the mean integrated density (MID, pixels/mm^2^) for treated versus untreated tumors. Graphs indicating the significant decrease in MID for COA6 and COA129 are shown, representing the significantly decreased viability (* *p* ≤ 0.05, *** *p* ≤ 0.001, treated vs. untreated). (**B**) Representative fluorescent photomicrographs of calcein AM stained COA6 untreated (top left panel) and treated (top right panel), scale bars represent 2 cm. COA6 microtumors were stained using immunohistochemistry for HSV (bottom panels). There was no HSV staining detected in the untreated microtumor (bottom left panel), but M002-treated microtumors showed positive staining for HSV (brown staining, white arrow, bottom right panel), scale bars represent 100 µm. (**C**, **D**) Media surrounding the microtumors were collected to assess for the presence of M002 and mIL-12 in the tumor microenvironment. Neither M002 nor mIL-12 was detected in the media of any COA129 microtumors, but there was a significant amount of (**C**) M002 progeny virions and (**D**) soluble mIL-12 detected in the media surrounding the COA6 microtumors (** *p* ≤ 0.01, *** *p* ≤ 0.001, treated vs. untreated). A Student’s *t*-test was used for statistical analysis.

PDXs more accurately mimic the human cancer condition when compared to long-term passage cell lines and thereby may better predict the clinical response to treatment [11,13]. A major disadvantage of PDXs is their inconsistent growth rates in animals that render their use for in vivo experiments challenging [35]. To overcome that obstacle, we employed a novel 3D bioprinted model to create PDX microtumors. Investigators have demonstrated that the 3D model may better replicate the human condition than traditional two-dimensional monolayer culture methods [36]. COA6 and COA129 microtumors were printed using the BioX bioprinter (Cellink). After five days of growth, the microtumors were treated with a single intratumoral injection of M002 (10^7^ PFU/10 μL PBS) or PBS (10 μL, control). The dose of virus was chosen based on previous intratumoral injections in in vivo flank tumor models [16,18,22]. One week after treatment, the microtumors were stained with calcein AM to detect live cells. The amount and intensity of staining was analyzed and compared between microtumors treated with M002 and those treated with PBS. The mean integrated density (MID), which is the total pixels over total area, of the fluorescence was used as a measure of tumor viability. COA6 microtumors treated with M002 collectively had a significantly lower MID when compared to those treated with PBS (1610 ± 82 vs. 807 ± 102 pixels/mm^2^, M002 vs. PBS, *p* = 0.00084, Figure 4A, left panel). COA129 microtumors were affected in a similar fashion, with fewer viable cells following M002 treatment (2801 ± 328 vs. 1160 ± 355 pixels/mm^2^, M002 vs. PBS, *p* = 0.0146, Figure 4A, right panel). Representative fluorescent photomicrographs of control (top left panel) and treated (top right panel) COA6 (Figure 4B) microtumors are shown. Histologic sections of COA6 (Figure 4B) microtumors stained for HSV-1 demonstrate the presence of virus in the treated microtumors (bottom right panel, white arrows) and absence in the control microtumors.

Since the M002 was injected directly into the microtumors and not into the media, we determined if progeny virions could be detected in the media as indirect evidence of viral replication and to quantify the amount of M002 in the tumor microenvironment capable of infecting surrounding tumor cells. The media surrounding the microtumors was collected and viral titer plaque assays performed. At 120 h post injection, progeny virions were detected in the media (Figure 4C) of only the COA6 tumors. Next, we sought to quantify mIL-12 in the media of these microtumors. Similar to viral progeny, at 120 h the COA6 microtumors produced a significant amount of mIL-12 in comparison to the COA129 microtumors (Figure 4D).

## 4. Discussion

Current therapy for high-risk neuroblastoma consists of cytotoxic chemotherapy, surgery, radiation, myeloablative chemotherapy with stem cell rescue, monoclonal antibody, and maintenance treatment. Despite this aggressive regimen, nearly half of these children will suffer from refractory disease or tumor relapse [2]. Oncolytic viral immunotherapy has potential to combat this aggressive malignancy. Oncolytic viruses have few side effects and have proven successful in numerous malignancies [37,38,39]. Our lab has previously shown that oHSV M002, effectively killed long-term passaged neuroblastoma cells and improved survival in mice bearing these tumors [22]. To expand upon and provide further justification for transition to a clinical trial, we investigated the use of M002 in neuroblastoma PDXs. We have successfully established three neuroblastoma PDX cell lines [40], two high-risk (COA3 and COA6) and one low-risk, MS neuroblastoma (COA129).

Investigators have begun employing three-dimensional (3D) printing technology to bioprint microtumors in efforts to better replicate the human condition over traditional two-dimensional monolayer culture methods. Langer et al. showed that printed tumors demonstrated characteristics commonly seen with in vivo tumors including drug resistance, tumor heterogeneity, and distinct tumor microenvironments [36]. The use of 3D technology has been more widespread in the adult oncology literature [12,41] and has had only limited reports in pediatric oncology. Pediatric cancer researchers have printed SH-SY5Y neuroblastoma cells to create a neural network [42] and SK-N-BE neuroblastoma cells to study CAR-T cell therapy [43], but to our knowledge, no one has yet reported bioprinting neuroblastoma PDX cells. In the current investigations, we successfully created bioprinted microtumors of the COA6 and COA129 PDXs. These microtumors were utilized as a surrogate for in vivo animal studies to substantiate the in vitro findings as we, along with others, have demonstrated that findings from in vitro studies with PDX cells were not always replicated in the in vivo PDX model [14]. The 3D architecture of tumors is crucial in their response to therapies [44], and although bioprinted tumors cannot exactly replicate an in vivo environment, it was promising to see effects of M002 on 3D microtumors that were similar to the previous in vivo findings with tumors from long-term passage neuroblastoma cell lines [22]. Additionally, the 3D microtumors provided the opportunity to quantify the number of progeny virions and amount of mIL-12 present in the surrounding tumor environment which has not been possible using an in vivo model and has yet to be done with this virus. These discoveries will provide insight regarding mechanisms that may be leveraged to improve cytotoxicity of oHSV therapy, perhaps through infection of tumor cells distant from the primary site or through stimulation of the immune system.

Both in vitro and in the ex vivo 3D printed tumors, there was marked M002 infectivity of tumor cells, but less replication in the low-risk MS neuroblastoma PDX compared to the two high-risk PDXs. This finding was confirmed by the multi-step viral replication and mIL-12 ELISA assays. There was also a slight decrease in viral replication (Figure 2B) and mIL-12 production (Figure 2C) seen in the COA3 cells at later time points. We believe the findings in the COA3 and COA129 PDX cells are secondary to different mechanisms. Replication of this genetically engineered virus depends upon live cells for their intact DNA replication machinery and therefore, the more cells killed by M002 over time will result in fewer cells available for the virus to use to replicate. The viability studies showed that at 0.1 PFU/cell, only 57.8 ± 8.7% of the COA3 cells were alive at 72 h post infection compared to the COA129, where 88.1 ± 2.3% were viable at the same dose and timepoint. This finding led us to hypothesize that the decline in replication seen in the COA3 cells was due to a lack of viable cells, whereas the decline in COA129 was independent of tumor cell death. Nevertheless, M002 did decrease COA129 cell viability. HSV-1 can also activate an apoptotic cascade leading to cell death [45,46], without a substantial lytic cycle cellular burst which may explain the decrease in the COA129 microtumor viability and absence of virions present in the surrounding media. These observations led us to formulate several hypotheses for specifically the decreased replication in COA129 tumor cells.

An initial postulate for the differences in infectivity was related to the difference in the percentage of cells with viral entry receptors present. CD111 is the most efficient receptor for HSV-1 entry [28,47] and each PDX had varying levels of CD111 expression with COA129 having the smallest percentage of cells expressing CD111. Friedman et al. demonstrated in pediatric brain tumors that the sensitivity of tumors to M002 correlated with CD111 expression up to a certain level, but as noted in previous publications, the baseline expression of CD111 was a biomarker for M002 success [20,24]. These studies noted increased expression of CD111 in highly migratory and invasive tumors; thus, COA129 may have low CD111 expression due to its low-risk nature. Although the COA129 demonstrated less infectivity than both the COA3 and COA6, the COA3 tumors also had lower levels of CD111 and proved to be equally susceptible to M002 infection as COA6. Therefore, we believe receptor concentrations may only be a small factor contributing to the noted differences seen between high-risk and low-risk neuroblastoma PDXs.

Finally, we investigated STAT1 as a potential mechanism for variable M002 infectivity. STAT1 is a key participant in the interferon signaling cascade responsible for abrogating viral replication [32,33]. As noted, M002 is missing the γ_1_34.5 gene, which Bin He’s laboratory determined is instrumental for HSV-1 to evade a cell’s interferon response and is critical for HSV-1 replication [48,49]. In support of these findings, Ghonime et al. found that glioblastoma (GBM) PDXs tumors with increased STAT1 activity were more resistant to oncolytic virotherapy [50], and concordantly, Kurokawa et al. showed similar findings with sustained interferon signaling [51]. In the current study, we found that STAT1 expression was higher at baseline in the COA129 PDX compared to the two high-risk PDXs. Furthermore, STAT1 expression increased with M002 infection in the COA129 PDX, suggesting the presence of an intact antiviral response mechanism. The converse was observed in the COA3 and COA6 PDXs, in which STAT1 expression decreased with M002 infection. Other investigators have found that major histocompatibility complex (MHC) Class I antigen presentation, a downstream sequalae of STAT1 [52], is dysregulated in high-risk neuroblastoma, which could indicate the ability for increased viral replication over time as we saw with the two high-risk PDXs in the current study [53,54]. In contrast, MHC Class I antigen presentation is retained in MS neuroblastoma [53] which may account for decreased viral replication in the low-risk COA129 PDX. Additionally, based on a publicly available database, low-risk neuroblastoma patient specimens had statistically significantly more STAT1 expression as compared to high-risk neuroblastoma specimens (Figure S2). This difference makes M002 an even more attractive therapy for high-risk disease.

Discovering significant differences in STAT1 in these neuroblastoma PDXs is an important finding, as this protein is targetable, thereby having the potential to be exploited to further augment tumor responses to oncolytic virotherapy. Other investigators have demonstrated that interruption of JAK/STAT signaling affected viral replication. In malignant peripheral nerve sheath tumors, Ghonime and Cassady interrupted STAT signaling with the Jak inhibitor, ruxolitinib, and showed significantly increased oHSV replication and decreased tumor size in vivo [55]. Similar studies with ruxolitinib in malignant T-cell lymphoma [56] and non-small lung cancer cells [57] demonstrated improved viral replication and tumor cell killing by reovirus and vesicular stomatitis virus. Based on the current findings, our future investigations will focus on further validation of the role of STAT1 in conferring resistance to oHSV in neuroblastoma, and the utility of combining oHSV with small molecular inhibitors of STAT signaling.

A benefit of M002 is its ability to activate an immune response not only through the creation of a debris field, but also through the production of the immunostimulatory cytokine, mIL-12. The ability to study the immune system in conjunction with oncolytic viruses is a challenge and weakness for cancer immunotherapeutic studies, including this study, since it requires the use of syngeneic tumor models. With the advent of 3D bioprinting, it will be possible to study an immune response to PDX microtumors. Tang et al. demonstrated this by successfully supplementing 3D GBM bioprints with macrophages and documented the ability of these macrophages to incorporate into the bioprint tumor microenvironment [58]. Future directions for our studies will involve the incorporation of immune cell components, such as NK cells and/or cytotoxic T-cells, into the 3D bioprints to assess the anti-tumor immune response in response to an IL-12 expressing virus.

## 5. Conclusions

The use of virotherapies to treat neuroblastoma is still limited and to date, there continues to be only a single clinical trial of oncolytic herpes viruses for the treatment of neuroblastoma [9,10]. The humanized version of M002, M032, is currently being studied in clinical trials (NCT02062827, NCT05084430) for treatment of adults with recurrent brain tumors. The studies presented here expand upon previous work from our laboratory investigating M002 in neuroblastoma. However, the current investigations are novel in that they employed human PDXs from both high- and low-risk neuroblastoma tumors, which more closely resemble the true clinical condition, and utilized bioprinted microtumors as surrogates for in vivo studies that demonstrated decreased viability with M002 treatment. These findings provide critical validation of efficacy and justification for advancing this therapy to the clinical arena, especially for children with high-risk disease.

## Data Availability

The data presented in this study are made publicly available in the Gene Expression Omnibus (GEO, Accession GSE49710).

## Supplementary Materials

The following are available online at https://www.mdpi.com/article/10.3390/cancers14030762/s1, Figure S1: Full Western blot scans are provided and densitometry performed for Figure 3C. Following infection with M002, there is a decrease in the expression of STAT1 in COA3 (**A**) and COA6 (B) with increasing MOIs (0, 0.1, 1 PFU) of M002. Conversely, there is an increase in STAT1 expression in COA129 (C) with M002 infection. (D) β-actin was utilized as a loading control for all blots. Figure S2: High-risk neuroblastoma expresses less STAT1.Using the SEQC publicly available database, STAT1 expression was compared across human neuroblastoma tumors that were classified as high-risk and non-high-risk. High-risk tumors were more likely to have significantly lower STAT1 in comparison to non-high-risk tumors.
