# Supplementary material for: Targeting High-Risk Neuroblastoma Patient-Derived Xenografts with Oncolytic Virotherapy
Source: Cancers (Basel). 2022 Feb 1;14(3):762. doi: 10.3390/cancers14030762 (PMC8834037; doi:10.3390/cancers14030762)
Supplement: Supplementary file 1 [file cancers-14-00762-s001.zip › cancers-1547192-supplementary.pdf]

A

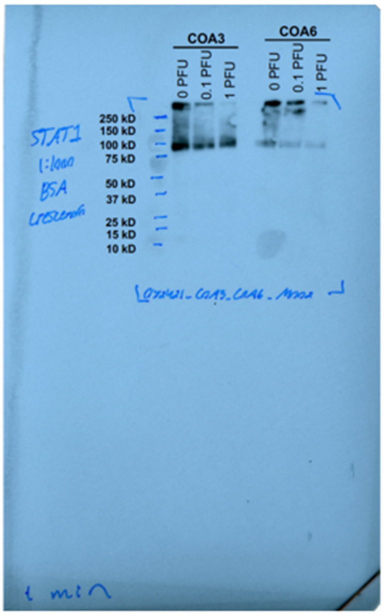

Lane	Ratio
COA3 STAT1 - 0 PFU	2.97
COA3 STAT1 - 0.1 PFU	2.05
COA3 STAT1 - 1 PFU	1.91

B

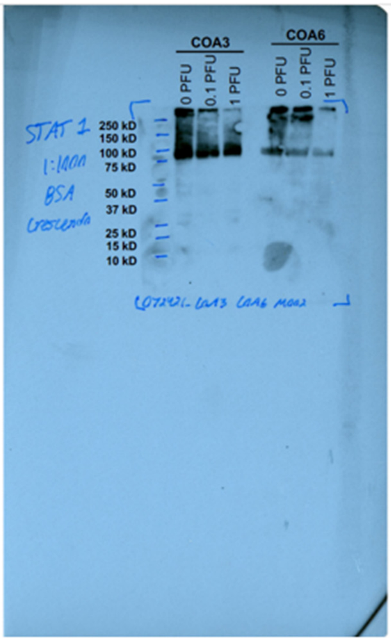

Lane	Ratio
COA6 STAT1 - 0 PFU	0.90
COA6 STAT1 - 0.1 PFU	0.63
COA6 STAT1 - 1 PFU	0.42

C

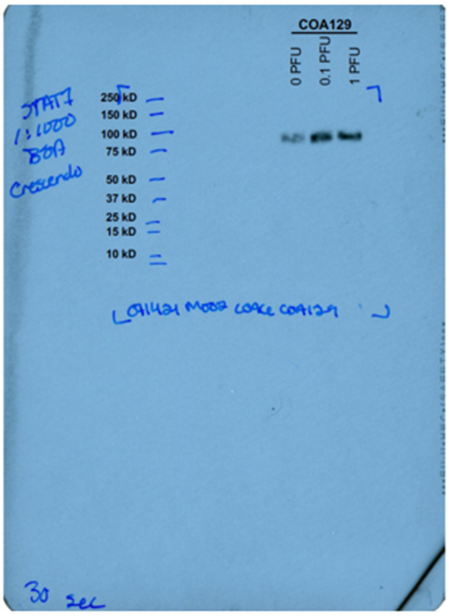

Lane	Ratio
COA129 STAT1 - 0 PFU	0.28
COA129 STAT1 - 0.1 PFU	0.58
COA129 STAT1 - 1 PFU	0.53

D

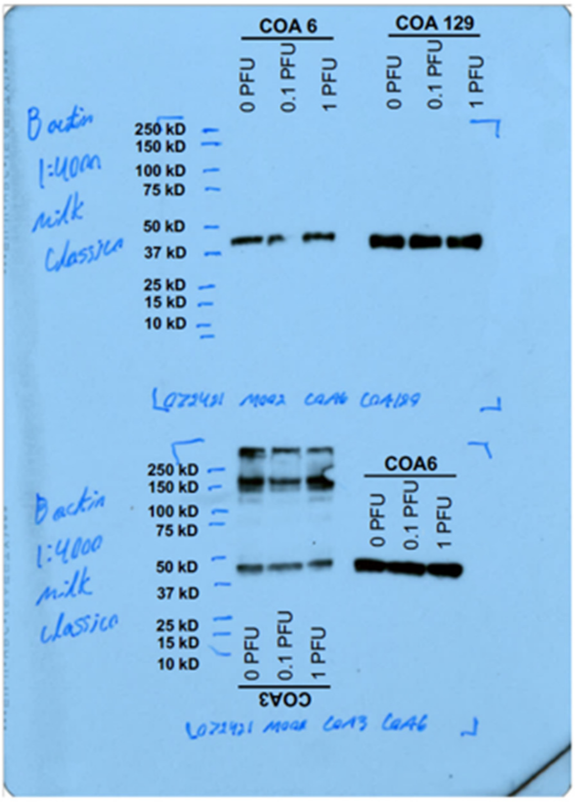

Figure S1. Full Western blot scans are provided and densitometry performed for Figure 3C. Following infection with M002, there is a decrease in the expression of STAT1 in COA3 (**A**) and COA6 (**B**) with increasing MOIs (0, 0.1, 1 PFU) of M002. Conversely, there is an increase in STAT1 expression in COA129 (**C**) with M002 infection. (**D**) β – actin was utilized as a loading control for all blots.

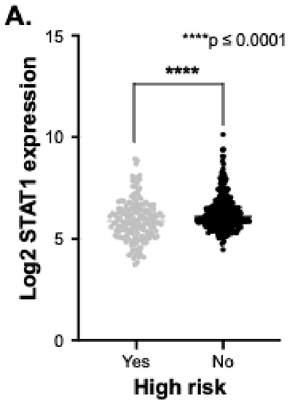

Figure S2. High-risk neuroblastoma expresses less STAT1. Using the SEQC publicly available database, STAT1 expression was compared across human neuroblastoma tumors that were classified as high-risk and non-high-risk. High-risk tumors were more likely to have significantly lower STAT1 in comparison to non-high-risk tumors.
